# Genetic Architecture of Charcoal Rot (*Macrophomina phaseolina*) Resistance in Soybean Revealed Using a Diverse Panel

**DOI:** 10.3389/fpls.2017.01626

**Published:** 2017-09-21

**Authors:** Sara M. Coser, R. V. Chowda Reddy, Jiaoping Zhang, Daren S. Mueller, Alemu Mengistu, Kiersten A. Wise, Tom W. Allen, Arti Singh, Asheesh K. Singh

**Affiliations:** ^1^Department of Agronomy, Iowa State University Ames, IA, United States; ^2^Department of Plant Pathology and Microbiology, Iowa State University Ames, IA, United States; ^3^Crop Genetics Research Unit, United States Department of Agriculture, Agricultural Research Service Jackson, TN, United States; ^4^Department of Botany and Plant Pathology, Purdue University West Lafayette, IN, United States; ^5^Delta Research and Extension Center, Mississippi State University Stoneville, MS, United States

**Keywords:** GWAS, *Macrophomina phaseolina*, soybean, breeding, resistance, charcoal rot

## Abstract

Charcoal rot (CR) disease caused by *Macrophomina phaseolina* is responsible for significant yield losses in soybean production. Among the methods available for controlling this disease, breeding for resistance is the most promising. Progress in breeding efforts has been slow due to the insufficient information available on the genetic mechanisms related to resistance. Genome-wide association studies (GWAS) enable unraveling the genetic architecture of resistance and identification of causal genes. The aims of this study were to identify new sources of resistance to CR in a collection of 459 diverse plant introductions from the USDA Soybean Germplasm Core Collection using field and greenhouse screenings, and to conduct GWAS to identify candidate genes and associated molecular markers. New sources for CR resistance were identified from both field and greenhouse screening from maturity groups I, II, and III. Five significant single nucleotide polymorphism (SNP) and putative candidate genes related to abiotic and biotic stress responses are reported from the field screening; while greenhouse screening revealed eight loci associated with eight candidate gene families, all associated with functions controlling plant defense response. No overlap of markers or genes was observed between field and greenhouse screenings suggesting a complex molecular mechanism underlying resistance to CR in soybean with varied response to different environments; but our findings provide useful information for advancing breeding for CR resistance as well as the genetic mechanism of resistance.

## Introduction

Soybean [*Glycine max* (L.) Merrill] is one of the most economically important crops due to its potential as an oilseed crop and major source of plant protein used for both livestock and human consumption. The United States (US) is responsible for 33% of the world production with a record 106.96 million ton in 2015, grown on 33.5 million ha and prices range from $296 to $351 per ton (http://www.usda.gov). However, production can be strongly compromised by abiotic stresses, pests, and pathogens (Hartman et al., 2015).

Charcoal rot (CR), a disease caused by *Macrophomina phaseolina*, can reduce both yield and seed quality (Smith and Wyllie, 1999). For economically important soybean diseases, it ranked among the top 10 in the US from 1996 to 2014, with an average of 1 million ton of yield loss according to the Extension and Outreach at the University of Illinois (extension.cropsciences.illinois.edu), and sixth in the top eight soybean producing countries in 2006 (Wrather et al., 2010). Charcoal rot is distributed worldwide in the tropics and sub-tropics, as well as in the US north central and southern regions (Wyllie, 1988), and *M. phaseolina* is known to infect over 500 plant species of economic importance including maize, sorghum (Adeyanju et al., 2015), and sunflower (Pawlowski et al., 2015). Annually, CR is a greater concern in the southern US due to frequent hot and dry conditions that tend to occur during important soybean developmental growth stages.

*M. phaseolina* is a soil- and seed-borne polyphagous fungus. The abundant production of minute black microsclerotia causes the infected plant tissues to blacken, and therefore, the disease is known as charcoal rot (Sarr et al., 2014). The fungus survives in the soil mainly as microsclerotia that are stimulated by root exudates to germinate and infect host plant material. Limiting soil moisture and higher air and soil temperature increases disease severity (28–35°C; Smith and Wyllie, 1999). The pathogen may move from infected roots to stems, clogging vascular tissues in the tap root (Kaur et al., 2012), and to seed, causing reduced germination, and seedling rots. Aboveground symptoms of CR appear after flowering (soybean growth stage R1), and are particularly evident in soybean fields at the R5 (beginning seed), R6 (full seed), and R7 (beginning maturity) growth stages (Wyllie, 1988). In severe situations, diseased plants may wilt and prematurely die.

Charcoal rot management strategies in soybean include cultural methods, seed-applied fungicide, and biological control, but these have not been effective or widely adopted and have provided limited control (Mengistu et al., 2015). In this scenario, genetic resistance may be the most feasible and sustainable method to manage CR (Mengistu et al., 2007). Complete resistance to *M*. *phaseolina* is not reported in any plant species, but identification of partial resistance has been reported in soybean, including moderately resistant cultivars, such as DT97-4290, used as a disease check standard (Paris et al., 2006; Mengistu et al., 2007, 2013; Twizeyimana et al., 2012; Pawlowski et al., 2015). However, investigations into commercially available germplasm and their general response to the fungus have not been widely performed.

Breeding for resistance is difficult because most diseases are quantitatively inherited and controlled by multiple genes; in such a scenario, methodologies that help to elucidate resistance mechanisms, and identify resistant genotypes contribute to increased success in breeding programs (St Clair, 2010). Primarily, breeding efforts have been focused on genetic entries from later maturity groups (MGs; e.g., MG IV and V), which are coincident with predominant CR regions in the southern US; however, the identification of CR in northern latitude soybean growing regions require research and breeding efforts in earlier maturity regions that plant early maturity varieties (typically MG III and earlier). Breeding efforts can be complemented with genome wide association studies (GWAS) as these serve a dual role of disease screening as well as identification of genetic markers and candidate genes.

Genome-wide association studies associate variation across the entire genome with phenotypes (Korte and Farlow, 2013) and are used to identify genetic variations of important traits including disease resistance (Iquira et al., 2015). These GWAS use high-density markers and a population of diverse individuals to provide greater mapping resolution than conventional methodologies, which enables the prediction or identification of putative causal genes, and reduce time and cost for the genetic dissection of traits (Song et al., 2013; Zhang et al., 2015b). In soybean, GWAS have been previously utilized to identify genes associated with resistance to Phytophthora root rot (Sun et al., 2014), soybean cyst nematode (Bao et al., 2014; Vuong et al., 2015), iron deficiency chlorosis (Mamidi et al., 2014; Zhang et al., 2017), sudden death syndrome (Wen et al., 2014; Zhang et al., 2015a,b), Sclerotinia stem rot (Bastien et al., 2014; Iquira et al., 2015; Zhao et al., 2015), and soybean aphid (Chang et al., 2016).

The aims of this study were to (1) identify new sources of CR resistance in 459 diverse soybean plant introduction (PI) lines from MG I, II, and III through field and greenhouse screening, and (2) conduct GWAS to increase our understanding of the resistance mechanisms and identify genetic markers associated with resistance that will contribute to the future selection of genotypes for breeding programs and genetic studies for CR resistance.

## Materials and methods

Two experiments were conducted to identify CR resistance sources and to locate candidate genes and markers related to resistance. The first experiment was a field screening using root and stem severity (RSS; Mengistu et al., 2007) to classify genotypes for resistance. The second experiment was a greenhouse screening utilizing the cut-stem methodology (Twizeyimana et al., 2012) to classify genotypes for resistance based on the area under the disease progress curve (AUDPC).

### Plant material

A collection of 459 soybean PI lines, spanning MG I to III, was obtained from the United States Department of Agriculture (USDA) Soybean Germplasm Collection. Two breeding lines (DT97-4290 provided by the USDA (Paris et al., 2006), and H3LER11017-00-0238 provided by Pioneer) were used as moderately resistant checks, and two others (Pharaoh provided by the USDA (Schmidt et al., 1993), and H3LER11022-00-0037 provided by Pioneer) were used as susceptible checks.

### *Macrophomina phaseolina* isolate and culture maintenance

An isolate of *M. phaseolina* collected from an Iowa soybean field in 2013 (D. Mueller lab, ISU) was used for all associated studies as outlined below. This isolate will be available to researchers with appropriate permits. From the plate of mycelium obtained from Dr. Mueller's lab, a mycelial plug transfer was made to create inoculum on PDA. We inoculated the fungus onto susceptible plants, then re-isolated the fungus to inoculate the soybean plants (field and greenhouse experiments in this study). The fungus had the same morphological characteristics as the original plate as well as showed similar symptoms on the susceptible plant inoculations. We completed Koch's postulates with the re-isolated pathogen (to inoculate susceptible soybean plants) to confirm its pathogenicity.

The inoculation method was adapted from Mengistu et al. (2007). Sorghum (*Sorghum bicolor* L.) seed (400 mL by volume) was soaked for 24–48 h in 4 L of distilled water. The liquid was decanted, and seed were equally divided and put into autoclavable bags. The autoclave cycle consisted of 121°C for 30 min, and samples were autoclaved twice. The autoclaved sorghum seed were put in bags containing 1.8 kg of sorghum grain and 1-week-old culture plugs of *M. phaseolina* (grown on PDA) were placed into each bag to inoculate the sorghum at a rate of 1 plate of fungus per 1.8 kg of seed. The bags were closed and incubated at 30°C for 2 weeks while periodically shaking the bag. After 2 weeks, the sorghum seed were completely colonized by the fungus and were darkened with microsclerotia. These seed were removed from the sealed bags to allow air drying, and were then stored in sealed plastic containers at 4°C until use.

### Field experiment

Four hundred sixty five soybean genotypes including 459 PI lines, four maturity checks (MN1410, LD02-4485, IA3023, and IA4005), one moderately resistant check (H3LER11017-00-0238, provided by Pioneer), and one susceptible check (H3LER11022-00-0037, provided by Pioneer), were grown near Muscatine, IA, in 1.52 m long single rows with 0.76 m row to row distance and 0.91 m alleyways. Maturity and disease checks were spaced every 100 and every 50 entries, respectively. Genotypes were arranged in a randomized complete block design (RCBD) with three replications. During field planting, the planter was calibrated to apply 3 g of charcoal rot-infected sorghum seed per linear 0.3 m in furrow with the soybean seed at a rate of 8 seeds/0.3 m using a 4-row planter (Almaco Company, Nevada, IA).

**Figure 1:**
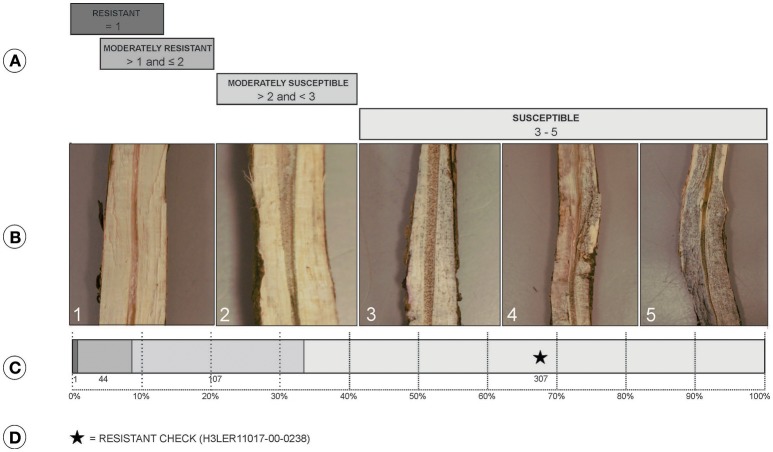
**(A)** Classes of charcoal rot resistance where resistant (values of 1), moderately resistant (values >1 and ≤2), moderately susceptible (values >2 and <3), and susceptible (values 3–5). **(B)** Split stem showing symptoms associated with the scores: 1, no microsclerotia visible in vascular tissue; 2, very few microsclerotia visible and vascular tissue is not discolored; 3, microsclerotia partially covering the vascular tissue and there is minimal discoloration; 4, numerous microsclerotia in the tissue and also visible under the outside epidermis, and discolored vascular tissue; 5, darkened vascular tissue due to high numbers of microsclerotia both inside and outside of the stem. **(C)** Distribution of the 459 PI lines and checks for resistance classification. **(D)** Class of location of the resistant check in accessions distribution.

Stem collection and charcoal rot ratings were based on the evaluation of root and stem severity rating (RSS; Mengistu et al., 2007). For each replication, five plants of each genotype were randomly harvested between the R7 and R8 growth stage from each row. Stem and top of the tap root portion of each plant was obtained by gently uprooting each plant and clearing it of the soil and other debris. Each plant stem was longitudinally split using a sharp knife and ratings were given on a scale of 1–5 (Figures 1A,B).

### Greenhouse screening

A total of 463 soybean genotypes were evaluated including 459 PI lines and four checks, two resistant and two susceptible checks repeated twice per block. The genotypes were arranged in a randomized complete block design (RCBD) with four replications. Two plants of each genotype in cups made for an experimental unit in each block. Plants were grown in 0.24 L styrofoam cups, with holes in the bottom for drainage, filled with soilless mix (Sunshine Mix, LC1; Sun Gro Horticulture Inc., Agawam, MA) and topped with pellets of slow-release fertilizer (Osmocote Plus 19-9-12, 23 g per cup) spread over the surface of each cup. Seeds were over sown, three seeds per cup, in each pot and thinned to two plants 10 days post-emergence. All experiments were conducted in a greenhouse maintained at 30°C day and 22°C night temperatures, and the room was supplemented with high-pressure 400 W sodium lights to ensure the 16-h photo period. Plants were watered manually to avoid plant wilting. The cut-stem inoculation technique was used to classify genotypes for resistance (Twizeyimana et al., 2012). Briefly, soybean plants were grown to the V2 growth stage and a razor sharp blade was used to cut 40 mm above the unifoliate node. From the growing margin of a 4-day old culture of *M. phaseolina* on PDA, a mycelial plug was obtained using a 200 μL pipette tip (Fisher Scientific). The pipette tip with mycelial plug was immediately placed over the stem cut by the razor blade and ensuring the agar was in embedded in the stem. Ratings were based on the recorded measurements of lesion length. The details of measurements are provided in Pawlowski et al. (2015) with the modification that the length of cut-stem at the time of inoculation was 40 mm instead of 25 mm used in their publication. Measurements were taken the third day after inoculation (dai), and followed every 3 days, for a total of 5 ratings (3, 6, 9, 12, and 15 dai). The area under the disease progress curve (AUDPC) for each entry was calculated to estimate the disease resistance and select superior PI lines (Jeger and Viljanen-Rollinson, 2001). AUDPC was used for GWAS using greenhouse data.

### Statistical analyses

The model for the phenotypic trait was *Y*_*ij*_ = μ + *g*_*i*_ + *b*_*j*_ + *e*_*ij*_, where μ is the total mean, *g*_*i*_ is the genetic effect of the ith genotype, *b*_*j*_ is the block effect, and *e*_*ij*_ is a random error following *N*(0, $\sigma_{e}^{2}$). Broad sense heritability was calculated as *H*^2^ = $\sigma_{g}^{2}$*/[*$\sigma_{g}^{2}$ + $\sigma_{e}^{2}$*/r]*, where $\sigma_{g}^{2}$ is the genotypic variance, $\sigma_{e}^{2}$ is the error variance, and *r* is the number of replications. The estimation of variance components was performed by R software with all effects considered to be random.

### Genotyping and quality control

The SNP dataset of the association panel was prepared by a previous study using the Illumina Infinium SoySNP50K BeadChip and was retrieved from the SoyBase (https://soybase.org/; Sonah et al., 2015). Of the 42,180 SNPs available for the association panel, 60 SNPs failed to anchor to the reference genome sequence and were excluded from further analyses. Individual markers with missing rates >10% were omitted, and the remaining missing data were imputed using BEAGLE version 3.3.1 with default parameter settings (Browning and Browning, 2007, 2009). SNPs with a minor allele frequency (MAF) <5% after imputation were also omitted for further analyses. Finally, 35,683 SNPs were used for GWAS.

### Marker distribution and linkage disequilibrium estimation

The Glyma.Wm.82.a2 reference genome was used to obtain chromosome physical lengths (bp) through SoyBase (www.soybase.org) which were used to calculate genome-wide inter-marker distance and chromosome-wide densities. Pairwise linkage disequilibrium (LD) between markers was measured using the squared correlation coefficient (*r*^2^) between alleles with the R package, synbreed (Wimmer et al., 2012). The *r*^2^ was calculated separately for euchromatic and heterochromatic regions due to the variability of recombination. Only significant *r*^2^ values (*P* < 0.001), calculated according to Remington et al. (2001), were considered informative. The chromosomal distance where the average *r*^2^ dropped to half of its maximum value was used to estimate the LD decay rate of the population (Huang et al., 2010).

### Genome-wide association analysis

To minimize the effect of environmental variation, best linear unbiased predictors (BLUPs) of individual lines were calculated using the R package, lme4 (Bates et al., 2014) for further analysis. The association analysis was conducted by using the genome assessment and prediction integrated tool (GAPIT) R package as descried in previous studies (Zhang et al., 2010; Lipka et al., 2012). No population structure was involved in the mixed linear model as a covariant as suggested by the Bayesian information criteria test.

The threshold for significant associations was determined by the empirical significance level of *P* < 0.001. To access the empirical significance of SNPs, a total of 1,000 permutations of genome-wide association was performed as previously described (Zhang et al., 2015a). For each iteration, the phenotype values and kinship matrix (K) in the MLM remained unchanged while genotypes of each SNP were permuted. The threshold was set at the lowest *P-*value of the SNP-trait association that did not meet the empirical significance level.

### Prediction of putative candidate genes

Genes annotated in Glyma1.1, Glmy1.0, and NCBI RefSeq gene models, available through SoyBase aligning to the Glyma.Wm.82.a2 reference genome (www.soybase.org), were used as the source of candidate genes. The significant SNPs in LD *r*^2^ > 0.7 with the peak SNP were clustered to form the candidate region of the quantitative trait locus (QTL). The peak SNP is defined as the SNP with the lowest *P*-value within the region defined above. The prediction of candidate genes resulted from the following priorities: (i) genes of known function related to soybean disease resistance, (ii) genes of known function as orthologs related to disease resistance in *Arabidopsis*, and (iii) genes pinpointed by the peak SNPs.

## Results

### Field screening

The RSS method, used in field screening, enabled identification of accessions with better disease resistance than the resistant checks (Figure 1). The mean score was 3.5 with a standard deviation of 1.05. PI379559D had the lowest score of 1.0 and classified as resistance as per the RSS classification (Paris et al., 2006; Mengistu et al., 2007). Twenty-six accessions had a score of 5.0, and the moderately resistant check, H3LER11017-00-0238, had a score of 4.0. Moderately resistant accessions present scores ranging from values >1 and ≤2, and for this experiment 44 accessions met these criteria. However, 90% of the accessions were classified as moderately susceptible and susceptible, with values >2 and ≤5.

### Greenhouse screening

Significant differences among the genotypes were observed (*P* < 0.001). The mean AUDPC value was 470 with a standard deviation of 124. PI603444A had the highest AUDPC value of 1,036, and PI567241 had the lowest AUDPC value of 270. The moderately resistant checks, DT97-4290 and H3LER11017-00-0238, had AUDPC values of 359 and 382, respectively, and the susceptible checks, Pharaoh and H3LER11022-00-0037, had a value range of 465 and 567, respectively.

**Figure 2:**
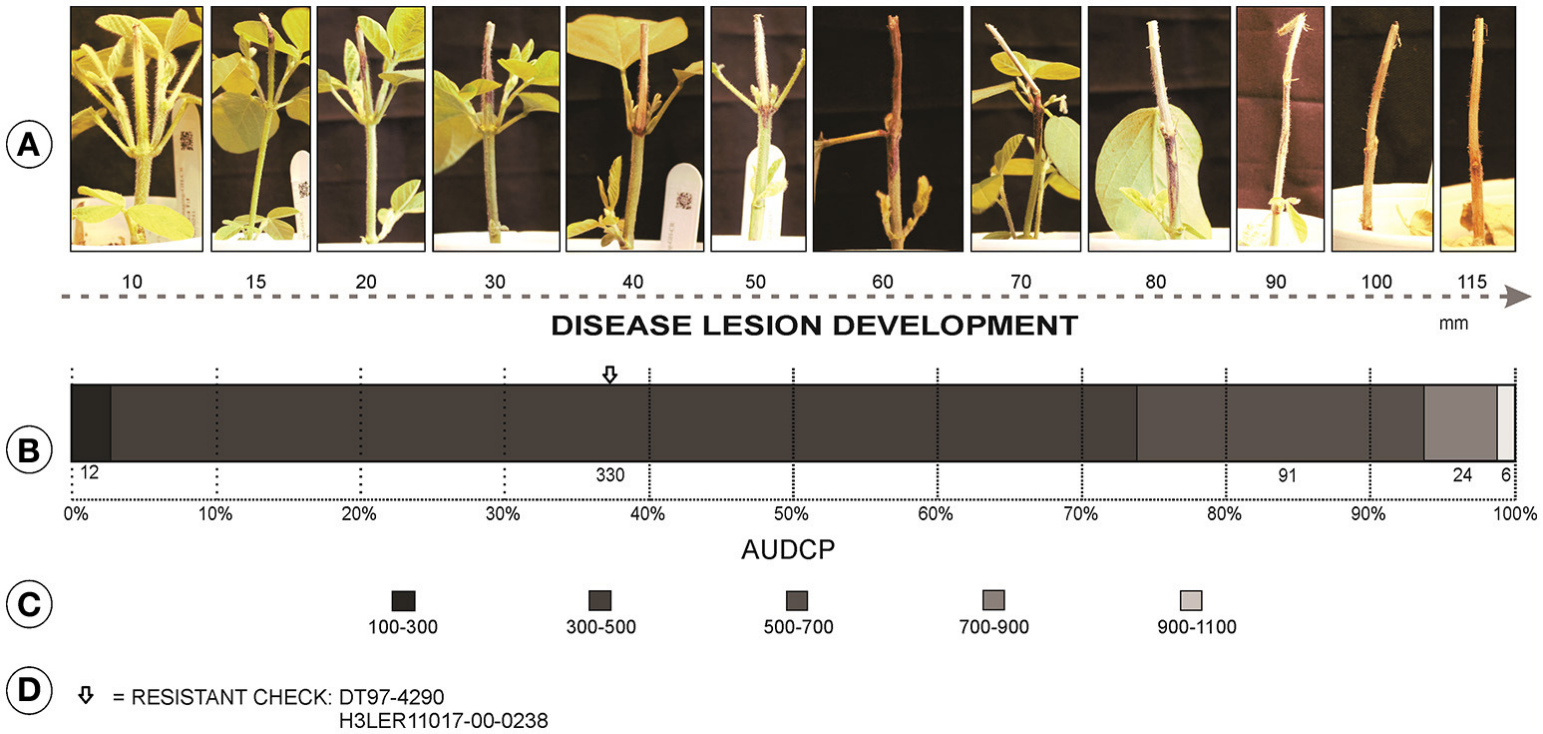
**(A)** Variation of disease lesion length (mm) for charcoal rot from disease screening in greenhouse. **(B)** Distribution of the 459 PI lines and checks for resistance classification based on AUDPC. **(C)** Classes of distribution of the AUDPC. **(D)** Class of location of the resistant checks in the distribution of the accessions.

Accessions with better resistance than the resistant checks were identified in the greenhouse screening (Figure 2). Among the 459 accessions, 51 (11%) exhibited AUDPC values less than the resistant checks. All accessions, even the most resistant, developed a lesion, which indicated that infection had occurred but that the plant was able to stop fungal development.

### Genotyping and quality control

For field screening, 155 (34%) accessions exhibited better CR resistance than the resistant check, and for greenhouse screening, 30 (7%) accessions were more resistant than the resistant checks. Table 1 shows the top 20 genotypes selected from maturity groups I–III for disease resistance from each experiment (field and greenhouse screening). Heritability values ranged from 0.60 to 0.58 for greenhouse and field screening, respectively (Supplementary Table 1). The field and greenhouse screening had a low but significant negative correlation (*r*_*fg*_ = −0.12). Among all the accessions selected for the field and the greenhouse, 22 (5%) were common between field and greenhouse experiments (Table 2) showing a good level of resistance in both and included accessions PI379559D and PI603594 with the lowest RSS and AUDPC scores, respectively.

**Table 1:** Genotypic value (GV) and maturity group (MG) of the top 20 plant introduction lines selected for charcoal rot resistance that were better than the resistant checks, variation of GV for low and high ratings, and GV for the resistant checks for field and greenhouse screening for charcoal rot resistance.

**Field**			**Greenhouse**		
**Accessions**	**GV**		**Accessions**	**GV**	
Lowest disease rating	2.1		Lowest disease rating	351.2	
Highest disease rating	4.3		Highest disease rating	806.2	
**Disease resistant checks**	**GV**		**Disease resistant checks**	**GV**	
DT97-4290	n/e		DT97-4290	392.2	
H3LER11017-00-0238	4.0		H3LER11017-00-0238	427.0	
**Top 20 accessions**	**MG**	**GV**	**Top 20 accessions**	**MG**	**GV**
PI379559D	III	2.1	PI567241	II	351.2
PI167240	III	2.1	PI549064	II	352.2
PI458507	III	2.1	PI379559D	III	356.6
PI461509	I	2.1	PI091725	II	360.2
PI538389	III	2.1	PI471899	III	360.9
PI189958	II	2.2	PI603594	II	361.2
PI091091	II	2.2	PI567277	II	362.1
PI548316	III	2.2	PI088497	I	362.6
PI549056	II	2.2	PI437462A	II	362.7
PI437377	III	2.2	PI504497	II	363.6
PI476911	II	2.2	PI567774B	III	364.9
PI538377	III	2.3	PI084973	III	366.8
PI578376	II	2.3	PI361090	I	371.2
PI091102	II	2.3	PI079694	I	371.9
PI091349	III	2.3	PI232989	II	372.1
PI479711	II	2.3	PI096322	III	372.9
PI578363	II	2.3	PI404169B	III	374.4
PI588008A	III	2.3	PI567250B	III	375.0
PI603594	II	2.3	PI574478B	II	379.6
PI092683	II	2.4	PI467307	I	379.7

**Table 2:** Selection of soybean genotypes with better charcoal rot resistance than the resistant checks for both field and greenhouse experiments using 459 plant introduction accessions from the USDA germplasm bank.

**Accessions**	**MG**	**Field rating**	**Greenhouse rating (AUDPC)**
PI567241	II	3.6	351.2
PI549064	II	3.0	352.2
PI379559D	III	2.1	356.6
PI091725	II	2.8	360.2
PI471899	III	2.9	360.9
PI603594	II	2.3	361.2
PI437462A	II	3.8	362.7
PI504497	II	3.8	363.6
PI567774B	III	3.8	364.9
PI084973	III	2.5	366.8
PI079694	I	3.9	371.9
PI232989	II	3.4	372.1
PI096322	III	3.2	372.9
PI404169B	III	3.6	374.4
PI567250B	III	3.5	375.0
PI574478B	II	3.2	379.6
PI467307	I	3.3	379.7
PI578499A	II	2.7	380.6
PI423870	II	3.2	383.4
PI227558	II	3.6	391.2
PI458307A	III	3.4	391.5
PI458506	II	2.8	391.5
**Disease resistant checks**	**Field**		**Greenhouse**
DT97-4290	n/e		392.2
H3LER11017-00-0238	4.0		427.0

### Genome-wide association analysis and prediction of putative candidate genes

A total of 19 SNPs associated with charcoal rot resistance were identified across chromosomes 4, 14, and 18 for field experiments, and chromosomes 6, 8, 9, 12, 18, and 20 for greenhouse experiments (Supplementary Figure S1). Based on the results of GWAS and genes annotated in SoyBase (www.soybase.org), four putative candidate genes were predicted for four of the six loci associated with field RSS scores. They are orthologs of *Arabidopsis* genes related to stress response, antimicrobial properties, and cell wall functions (Table 3). For the greenhouse AUDPC, a total of 11 putative candidate genes were predicted for eight of the 30 loci associated with the trait. Three of them are orthologs of *Arabidopsis* genes related to cell defense components and disease plant resistance proteins (Table 3).

**Table 3:** SNPs significantly associated with charcoal rot resistance through GWAS and predicted candidate genes for field and greenhouse screening.

**Chromosome**	**SNP**	***p*-value**	**Gene**	**Annotation**	**Ortholog**	Ortholog function^*^
**FIELD**						
4	*ss715588228*	1.45E-04	*Glyma.04g053100*	Universal stress protein family (USP); Leucine-rich repeat receptor-like protein kinase	AT5G12000.1	Response to stress
14	*ss715618004*	5.87E-05	*Glyma.14g002000*	Multi Antimicrobial Extrusion (MATE); Multidrug resistance protein	AT2G38330.1	Transmembrane transport
18	*ss715631906*	4.63E-05	*Glyma.18g248100*	Cyclophilin (CyPs)	AT4G17070.1	Response to oxidative stress
18	*ss715631726*	3.18E-04	*Glyma.18g228600*	LysM domain	AT5G62150.1	Cell wall macromolecule catabolic process
**GREENHOUSE**						
6	*ss715593307*	4.20E-06	*Glyma.06g176100*/*Glyma.06g176200*	Cytochrome P450	AT3G48310.1	Abiotic and biotic stress response
8	*ss715601990*	2.67E-06	*Glyma.08g306800*/*Glyma.08g306900*	Glutathione S-transferase C-terminal domain (GST)	AT3G20410.1/AT4G25130.1	Cell defense/protection from oxidative stress
8	*ss715602087*	1.95E-06	*Glyma.08g315900*/*Glyma.08g316500*	Peptide methionine sulfoxide reductase (PMSR)/CDPK calmodulin-domain protein kinase isoform 9	AT5G61460.1/AT5G62420.1	Chromosome structure/oxidation reduction
9	*ss715604575*	7.46E-05	*Glyma.09g230300*	Leucine Rich Repeat (LRR)	AT5G61480.1	Vascular tissue development
12	*ss715612760*	3.73E-08	*Glyma.12g216200*	Terpene synthase (TPS)	AT5G23960.2	Sesquiterpenes generator
12	*ss715613120*	4.04E-05	*Glyma.12g006300*	Leucine Rich Repeat (LRR)	AT5G61480.1	Vascular tissue development
18	*ss715632099*	2.93E-04	*Glyma.18g262800*	AP2 domain	AT5G19790.1	Encondes an ethylene response factor
20	*ss715638424*	1.26E-06	*Glyma.20g197000*	AP2 domain	AT4G13620.1	Encondes an ethylene response factor

* The Arabidopsis Information Resource (https://www.arabidopsis.org/).

Field screening identified four putative candidate genes (Table 3). The one, *Glyma.04g053100*, on chromosome 4 was identified at 6.4 kb downstream of *ss715588228*. *Glyma.04g053100* belongs to a universal stress protein family (USP) protein and is homologous to the *AT5G12000.1* that encodes a Leucine-rich repeat receptor-like protein kinase response to stress (Table 3). On chromosome 14, the candidate gene *Glyma.14g002000* was identified at 6.9 kb away from the peak SNP *ss715618004*. It encodes a multidrug resistance protein and is homologous to the *AT4G38380.1*, which encodes a transporter of plant metabolites related to defense signals (Table 3). On chromosome 18, two gene candidates, *Glyma.18g248100* and *Glyma.18g228600*, were found at the proximity of the peak SNP *ss715631906* and *ss715631726*, respectively. The former is homologous to *Arabidopsi*s *AT4G17070.1* encoding a peptidyl-prolyl cis-trans isomerase and is related to the response to oxidative stress and pathogen infection. The latter produces a protein containing the LysM domain, which is related to cell wall catabolism and fungal pathogen defense in *Arabidopsis* (Table 3).

Greenhouse study identified eight loci across six chromosomes (Table 3). On chromosome 6, two candidate genes were identified at 24.5 and 14.8 kb upstream of the peak SNP *ss715593307*. Both encode a Cytochrome P450 protein involved in biosynthetic reactions including defensive compounds such as terpenoids. On chromosome 8, two loci were identified, each with two candidate genes. *Glyma.08g306800* and *Glyma.08g306900* at the proximity of *ss715601990* encoding a Glutathione S-transferase, proposed to be involved in the synthesis of stress-related proteins and defense components. The second locus was targeted by two SNPs in high LD (*r*^2^ > 0.7). Two genes located at 21.3 kb downstream and 35.8 kb upstream of the leading SNP *ss715602087* were identified. *Glyma.08g315900* and *Glyma.08g316500* encode a peptide methionine sulfoxide reductase (PMSR) and a Calmodulin-domain Protein Kinase (CDPK), respectively, and both are related to abiotic and biotic stress responses. On chromosome 9, *Glyma.09g230300* was identified 15.6 kb downstream of the lead SNP *ss715604575*. It encodes a leucine rich repeat (LRR) protein that is involved in disease resistance mechanisms. The candidate gene, *Glyma.12g006300*, was identified in a region consisting of 16 significant SNPs in high LD (*r*^2^ > 0.7) on chromosome 12. It also encodes a LRR type protein and is related to disease defense components and stress related responses. On chromosome 18, putative candidate gene *Glyma.18g262800* is located 2.9 kb downstream of the peak SNP *ss715632099* and encodes an AP2 domain-containing inductor of ethylene-responsive element (ERE) that is associated with plant disease defense. Similarly, *Glyma.20g197000* encoding an AP2 domain-containing protein was found at the close proximity of the peak SNP *ss715638424* on chromosome 20.

## Discussion

Charcoal rot is an important fungal disease; however, limited information is available on the resistance of earlier maturity soybean accessions to this disease in the US. From both field and greenhouse screenings in the current research, new sources of charcoal rot resistance were identified for MGs I to III among the accessions that performed better than or similar to the resistant checks. Pawlowski et al. (2015) reported three PI lines from maturity groups I, II, and III exhibiting partial resistance to *M. phaseolina* in a greenhouse screening of 81 genotypes using the cut-stem technique; however, there was no overlap with the genotypes from this study. The present study identifies previously unreported sources of charcoal rot resistance from early MGs (I, II, and III), contributing to the development of CR resistant cultivars adapted to northern soybean growing regions in the US, and complements the screening of soybean genotypes in the later maturities (MG IV and beyond; Mengistu et al., 2007). The identification of these accessions, together with the heritability value, reinforces the existence of disease expression repeatability and usefulness to breeding programs. Utilization of field and greenhouse testing for charcoal rot resistance allowed the identification and selection of soybean accessions with better disease resistance than the resistant check in both field and greenhouse environments.

The field and greenhouse screening allowed the comparison of different environments in an attempt to understand the mechanisms of resistance involved in each and to verify correlation between expression of resistance in field and greenhouse environments. The correlation between field and greenhouse experiments was significant, but showed a low negative value (*r*_*fg*_ = −0.12). Charcoal rot resistance has been screened primarily through field evaluations (Bristow and Wyllie, 1984; Pearson et al., 1984; Smith and Carvil, 1997; Mengistu et al., 2007). However, with the development of new inoculation methodologies, some screenings have been evaluated in controlled environments of greenhouse and growth chamber (Bristow and Wyllie, 1984; Surrette et al., 2006; Twizeyimana et al., 2012). No researchers screened plants under both field and greenhouse conditions, although Twizeyimana et al. (2012) had reported a comparable result for the cut-stem methodology with previously reported field results. It is important to highlight that these comparable results compared 16 PI lines pre-screened for resistance and did not represent a true correlation of a heterogeneous population consisting of variable resistance responses.

The lack of correlation between field and greenhouse experiments for disease assessment is commonly reported in literature (Kim and Diers, 2000; Hoffman et al., 2002; Hartman et al., 2014). Environmental factors, including rainfall and temperature, and plant maturity have major effect on the severity of CR and must be considered when correlating greenhouse and field studies that evaluate a diverse maturity set of soybean accessions (Pawlowski et al., 2015). Several issues with greenhouse and field disease experiments, such as control of environmental conditions, physiological differences between genotypes, resistance mechanisms, uniform concentrations of inoculum, and inoculation location on the plant should be considered to help standardize and correlate future research efforts. Plants in field tests are indirectly inoculated by placing inoculum with the seed during planting, and the initial inoculum content of soil is generally not quantified. Furthermore, the rating methodologies for field and greenhouse screening may be of influence because one rating is subjective based on visual symptoms (RSS), and the other is directly quantified by measuring the extent of necrosis (cut-stem method). Another issue is related to the plant growth stage and physiology. A plant's response to abiotic and biotic stresses involves complex signaling pathways that depend on numerous genes, proteins, and metabolites, reflecting in differences in the resistance mechanism, which may also vary across life stages (Radwan et al., 2013). In the current studies, disease evaluation occurred at the V2 growth stage in the greenhouse screening, and R7 growth stage for the field screening. Response differences due to artificial wounding (in cut stem method) and growth stage may relate to the expression of different genes for disease reaction mechanisms, and environmental conditions for field settings can generate genotype by environment interactions.

Few accessions showed better performance than the checks for both field and greenhouse screening (22 accessions from 459), and need careful development of strategies for selection. Although greenhouse screening is considered faster and less laborious, the correlation between resistance observed in the greenhouse vs. in field screening is not consistently related. Therefore, field screening, which better represents the final environment for crops, should still be considered in the screening process. However, it should be considered that the available methods for CR assessment are few and new, and still under processes of improvement. In this scenario, further investigation for field and greenhouse methods which correlate well in classifying genotypes for CR resistance are needed and recommended to enable secure and reliable selection and classification of resistant genotypes. The interest in greenhouse screening still exists because it is less time and resource intensive'; however, only after a greenhouse screening methodology that is positively correlated with field expression is developed, can controlled (indoor) condition screening be effectively used as a pre-screening prior to a more thorough field screening.

*M. phaseolina* is known as a generalist because no specific resistance genes are reported in any of its hosts. There is no complete resistance reported for this pathogen among the host species probably because resistance is quantitatively controlled. The current research is the first study to our knowledge to use a genomic tool (i.e., GWAS) to identify genes related to CR resistance in soybean in order to better understand the pathway of genetic expression and association with resistance. Five gene families were associated with the resistance response for the field screening. Among them, the USP family and the cyclophilins (CyPs) are both related to stress response. USPs are small cytoplasmic proteins which have been related to pathogen-plant interaction. Lenman et al. (2008) reported that the USP were the first plant protein to phosphorylate in response to the inoculum presence when inoculating *Arabidopsis* plants with *Phytophthora* spores. CyPs are ubiquitous proteins, that are induced by various stresses including temperature (low or heat shock), light, salt stress, physical injury, and pathogens (Marivet et al., 1994). In diseased plants, cyclophilin may function as a “chaperon-like molecule” that decrease the risk of proteolytic degradation or help avoid accumulation during stress.

One of the genes identified is a characterized member of the multi antimicrobial extrusion (MATE) protein which has been reported to be involved in many physiological functions. Among these functions are alkaloid accumulation, flavonoid accumulation, and plant hormone signaling, all directly related to defense responses against herbivores and pathogens (Shitan, 2016). MATE transporters are associated with plant disease resistance, transporting compounds such as salicylic acid (SA) that plays an essential role in plant innate immune signaling and in disease resistance. Initiation of plant antimicrobial defenses in response to attempted microbial infection relies on a molecular dialog between the interacting organisms. *LysMs* genes have been associated in the recognition of carbohydrate patterns commonly related with microbial surfaces and in microbial infection immunity (Willmann et al., 2011). Wan et al. (2008) demonstrated that plant cells can perceive chitin fragments that lead to gene induction and defense responses by a *LysM receptor*-*like kinase1* (*LysM RLK1*) in *Arabidopsis thaliana*, indicating that *LysM RLK1* is essential for chitin signaling and plant innate immunity.

For the greenhouse screening, eight gene families were related to the mechanism of response to CR resistance. Among them, a cytochrome P450 was identified on chromosome 6, which is widely reported for its function in plant defense, including soybean cyst nematode and soybean rust (Irmisch et al., 2015; Wan et al., 2015; Langenbach et al., 2016). Three candidate genes were proposed on chromosome 8. Glutathione S-transferases (GSTs) has been connected in reactions linked to secondary metabolism and response to pathogen, including its importance in regulation of jasmonic acid (Han et al., 2013) and has been reported in multiple species, for example, *Populus tomentosa* (Liao et al., 2014) and *Lilium regale* Wilson (Han et al., 2016). Peptide methionine sulfoxide reductase (PMSR) acts as an antioxidant, repairing proteins damaged from oxidative stress and a novel defensive role against attack by pathogens *Phytophthora capsici* and *P. infestans* on pepper (*Capsicum annuum*) via regulation of the cellular levels of reactive oxygen and defense-related genes (Oh et al., 2010). Calcium-dependent protein kinases (CPKs or CDPKs) are regulated by various external stimuli including temperature stress (heat or cold), water stress, salinity, wounding, and biotic stress. NtCDPK2 has been reported to be activated by race-specific pathogen elicitation (Cf-9/Avr9) and abiotic stress in tobacco (Romeis et al., 2000). On chromosome 12 a strong association was detected and leucine rich repeat (LRR) and Terpene synthase (TPS) related candidate genes were identified. LRR proteins plays a crucial role in the plant's defense against pathogens, for their “immune” functions and recognition of non-self-molecules, including from GWAS in soybean (Li et al., 2016). Genome-wide identification and evolutionary analysis of LRR genes in soybean reported 467 putative LRR-receptor like kinase (LRR-RLK) genes in the soybean genome (Zhou et al., 2016). Terpene synthase (TPS) act as signaling molecules that induce defenses against tissue damage from wounds and microbes infected sites. Recent research demonstrated a terpene synthase 24 (OsTPS24) encoding a jasmonate-responsive monoterpene synthase that produces an antibacterial γ-terpinene against pathogens in rice (Yoshitomi et al., 2016). On chromosome 18 and 20, the APETALA2/ethylene-responsive element binding protein (*AP2*/*EREBP*) plays various roles in plant growth and development, and in response to stresses as pathogen infection, drought, temperature, and salinity (Zhang et al., 2013; Tang et al., 2016). It has been reported that the ethylene pathway was activated in response to the pathogen attack in several crop species (Dong et al., 2015; Wang et al., 2015; Agarwal et al., 2016; Hong et al., 2016).

Genome wide association studies have been reported as a very successful approach to investigating SNP-disease associations and deciphering the trait genetic architecture. The results reported in this study suggest that CR has a complex genetic architecture, with a polygenic background, classified as a quantitative trait. Due to its quantitative nature, information generated in this study is more suitable applied to genomic selection approaches rather than marker assisted selection (MAS), as MAS is inadequate for improving quantitatively inherited, i.e., polygenic traits. Genomic selection can improve genetic value estimations and selection accuracy for breeding for CR resistance. The information related to the candidate genes and markers reported are the first report for CR disease in soybean and require validation of marker-trait association in bi-parental mapping populations. Additionally, it is highly necessary that the genes are submitted to validation studies through gene silencing and expression studies.

It is important to highlight that CR disease is a disease commonly observed in the southern soybean production region of the US, especially during years when hot and dry conditions persist (Romero Luna et al., 2017). The disease has been spreading to the northern soybean growing regions of the US and have been reported in northern states including Iowa (Yang and Navi, 2005), Michigan (Baird et al., 2010), Minnesota (Elaraby et al., 2003), North Dakota (Bradley and Río, 2003), and Wisconsin (Birrenkott et al., 1984). More information about this disease and its evolution through the production regions is needed in order to develop resistant cultivars. Additional research is needed to determine if resistance mechanisms are the same or different in northern and southern adapted germplasm.

## Conclusion

The current study is the first effort to apply GWAS to understand and explain the genetic mechanisms underlying resistance to charcoal rot in soybean. We identified 5 and 8 loci for field and greenhouse screening, respectively, which were associated with candidate genes involved in controlling the plant defense response. However, the lack of overlap in significant SNP and candidate gene in field and greenhouse screenings, indicates that different mechanisms are involved in disease resistance expression in field and greenhouse. This research serves as a basis for the identification of candidate genes involved in CR resistance and unravels the complexity of this resistance. In the materials studied in our study, due to the prevalence of smaller effect loci controlling CR, genomic selection is an attractive strategy. The genetic entries identified in this paper will be useful for improving charcoal rot resistance in soybean.

## Author contributions

AKS and AS formulated research problem and designed the approaches, AKS and AS directed field efforts and phenotyping; SC and RC collected phenotypic data; SC, JZ, RC, AKS analyzed the data. All authors contributed to the preparation and development of manuscript.

### Conflict of interest statement

The authors declare that the research was conducted in the absence of any commercial or financial relationships that could be construed as a potential conflict of interest.
