# Supplementary material for: Genetic Architecture of Charcoal Rot (Macrophomina phaseolina) Resistance in Soybean Revealed Using a Diverse Panel
Source: Front Plant Sci. 2017 Sep 21;8:1626. doi: 10.3389/fpls.2017.01626 (PMC5613161; doi:10.3389/fpls.2017.01626)

Supplementary Table 1. Components of variance from (a) field and (b) greenhouse experiments including checks and PI accessions assessed for charcoal rot reaction.

Supplementary Table 1a. ANOVA and variance component analysis of the greenhouse experiment using AUDPC.

	Df	Sum Sq	Mean Sq	EMS	F value	Pr(>F)
Gen	462	28271361	61193	9124	2.49	<2e-16 ***
Rep	3	16487320	5495773		222.53	<2e-16 ***
Residuals	1383	34155997	24697	24697		

Supplementary Table 1b. ANOVA and variance component analysis of the field experiment using resistance score.

	Df	Sum Sq	Mean Sq	EMS	F value	Pr(>F)
Gen	464	1553	3.35	0.65	2.38	<2e-16 ***
Rep	2	115.9	57.93		41.11	<2e-16 ***
Residuals	1028	1448.6	1.41	1.41		

Supplementary Figure S1. Manhattan plots of GWAS for (a) field scores and (b) greenhouse AUDPC screening related to charcoal rot resistance in soybean. Negative log10- transformed P values of each SNP from a genome-wide scan by using mixed linear model (MLM) were plotted against the physical position on the soybean genome. The line indicated the significant threshold of P < 0.001, and the significant trait-associated SNPs were highlighted in red.

(a)

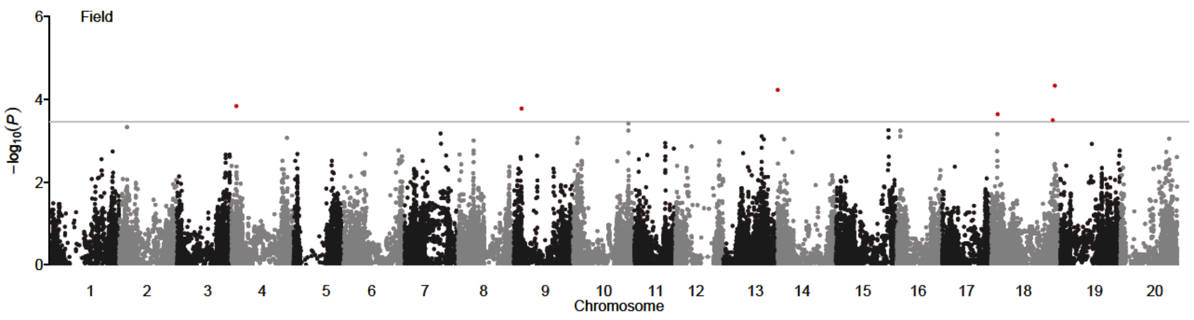

(b)

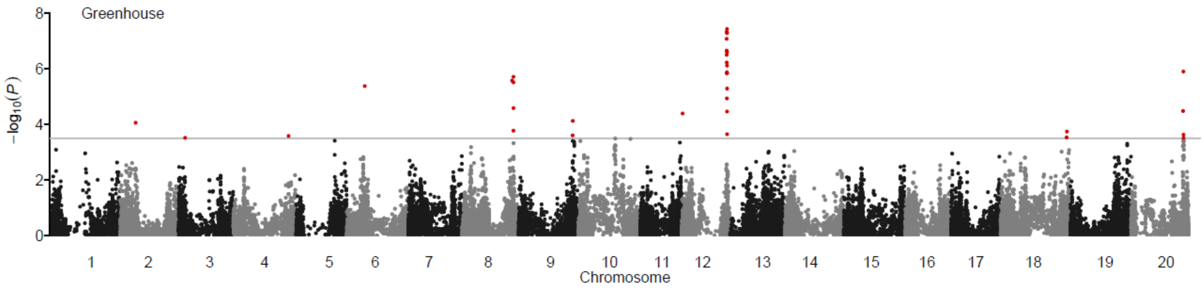

Supplement: Supplementary file 1 [file Image1.PDF]
